# Malaria Prevalence and Its Associated Risk Factors among Patients Attending Chichu and Wonago Health Centres, South Ethiopia

**Published:** 2016-12-21

**Authors:** Eshetu Molla Belete, Amanuel Bedane Roro

**Affiliations:** ^a^ Department of Medical Laboratory Sciences, College of Health Sciences and Medicine, Dilla University, Dilla, Ethiopia; ^b^ Laboratory Unit, Chichu Health Centre, Chichu, Ethiopia

**Keywords:** Malaria, Prevalence, *Plasmodium*, risk factors, Ethiopia

## Abstract

**Background:** There were about of 124 to 283 million cases of malaria with 367,000 to 755,000 deaths annually. This study aimed to assess the prevalence of malaria cases and associated risk factors among attendants at Chichu and Wonago health centers, South Ethiopia.

**Methods:** In this health institution based cross sectional study, 324 subjects, attendants from outpatient department who came for any kind of medical services, were included during May to June 2016. A blood film examination format and structured questionnaire were used for data collection. Peripheral blood samples were collected and the presence of malaria cases was observed microscopically. The collected data were analyzed by SPSS version 20.0.

**Results:** Malaria cases were detected in 91 (28.1%) of the participants with higher infection rate amongst (56.04%). The predominant *Plasmodium* species detected was *P. vivax* (52.75%) followed by *P. falciparum* (35.16%) and mixed malaria infection by both of the species (12.09%). Housing construction and not using of insecticide treated bed nets for the last 6 months were significantly associated with the risk of getting malaria. Individuals who had stagnant water in their compound were more likely to get malaria than those who did not (OR=1.87, 95% CI: 1.20, 2.76). Houses that had been sprayed with insecticide in the past 6 months were protected against malaria infection (OR=0.33, 95% CI: 0.11, 0.92). Moreover, bed net utilization was associated with a significantly lower risk of infection (OR=0.19, 95% CI: 0.09, 0.37).

**Conclusions:** Type of housing construction, not using bed net, insecticide spraying and residing near stagnant water were associated risk factors with malaria positivity in the study area.

## Introduction

Malaria is endemic throughout most of the tropics and is caused by the protozoan parasites of one of the four species of *Plasmodium*; *P. falciparum*, *P. vivax, P. ovale* and *P. malariae* and transmitted via the bites of female anopheles mosquito vectors^1,2^. Of these, *P. falciparum* and *P. vivax* are the most common^2^. From the estimated 1 million malaria deaths worldwide, 90% occur in Africa, killing mostly under 5 years of age children^3^, who account for 78% of all deaths^4^.

Malaria affects non-immune individuals in many parts of the African continent^5^. The control of the disease and its vectors in Africa is less successful because of the occurrence of antimalarial resistant parasites and insecticide resistant vectors, the shifting behavior of mosquitoes (from indoor to outdoor) as a result of frequent indoor insecticide sprays, lack of efficient infrastructure, shortage of trained manpower, lack of appropriate management, shortfalls in funding and inability to integrate different methods of control^6^.

Ethiopia is one of the seriously affected countries in sub-Saharan African countries, as malaria is the top ranking in the list of common communicable diseases in the country^7^. It is one of the leading causes of morbidity and mortality in Ethiopia^3^. Nearly 3/4 of the land is malarious, with malaria primarily associated with altitudes as high as 2500 meters and high risk with rainfall patterns above 100 mm (but peak during and just after the rainy season),^9^. According to the report of The Carter Centre^3^, approximately 55.7 million people in Ethiopia faced the risk of getting malaria, and approximately 80% of the 736 districts in the country are considered malaria endemic. Malaria transmission peaks bi-annually from September to December and from April to May in most areas of the country^10^.

Documenting the current epidemiologic status of malaria and identification of its risk factors can urge the decision makers to act timely to develop targeted interventions^11^. Major contributing factors such as housing type, house proximity to mosquito breeding sites or stagnant water, toilet facilities, and malaria preventive measures have been identified in different studies^12-14^. Other factors such as bed nets availability per household, individual’s age and residence altitude, as well as household wealth, temperature, peak monthly rainfall can affect malaria prevalence^15^.

The overriding objective of this study was therefore to determine the current prevalence of malaria cases and associated risk factors among the study subjects.

## Methods

The study was conducted in Chichu and Wonago health centres Gedeo Zone, Southern Nations, Nationalities and Peoples’ Region, among outpatient department attendants who came for medical services. Gedeo zone is 365 km away from Addis Ababa, the capital of Ethiopia. The health centres provide different medical services including malaria microscopic diagnosing services for inpatient and outpatient attendants. The two major malaria transmission periods in the area are from September to December and from April to June. The study was conducted from May to June 2016.

A health institution based cross-sectional study design was conducted among subjects attending the Chichu and Wonago health centers. Patients, attended for any medical services, were included in the study at their follow-up visits during the study period at outpatient department of the health centres. All individual members who have been living for more than six months, having history of fever during the past 24 h, and volunteer to participate in the study were enrolled. In addition, all individual members with anti-malarial treatment for the last 8 days were excluded from the study.

Sample size was calculated using the single population proportion formula, and prevalence was assumed to be 25.8% of the study conducted in Hadiya Zone health facilities, Ethiopia^16^, 0.05 precision with 95% confidence interval (CI), and 10% of non-response rate. Finally, 324 individuals were enrolled. For sampling method, the 324 sampled participants were distributed to the two health centres proportionate to the number of study subjects. These health centres were selected purposely classified as the catchment health facilities for malaria endemic districts. Estimated patients in both health centres (Chichu 162 participants and 162 study subjects from Wonago) from outpatient department were selected. Study subjects were randomly selected and all eligible attendants were invited to participate.

Before blood sample collection, the finger was cleaned with alcohol-moistened cotton. Then, a drop of blood, approximately 50 μL (capillary blood from fingertip) was collected. Two blood slides each composed of thick and thin films, were taken from each study subject according to the standard operating procedure^17^. Slides were labeled and air-dried horizontally in a slide tray in the field, and thin films were fixed with methanol after drying. Slides were stained with 3% Giemsa for 30-45 min at laboratory unit^18^. Blood slides were read and crosschecked at the laboratory unit in the health center for the positivity and negativity of the slides. All procedures were conducted by following the standard protocol of WHO. Then, the positivity of parasite was determined from thick smear preparations and species identification was carried out from thin smear. Forms were checked by the supervisors and inconsistencies were verified with the respondents. To ensure the validity of the slide test, all samples were re-examined by another independent experienced medical laboratory technologist. Finally, kappa statistics was performed to check the reliability between the two results (kappa=0.926).

For associated risk factors, the survey questionnaires were used, based on the malaria indicator survey household questionnaires modified for the local conditions, filled by the participants^19^. The questionnaire was administered to volunteers from whom the blood film was drawn according to the time schedule of each subject.

The collected data were entered and cleaned using Epi INFO vision 3.1. After cleaning, the data were transported to SPSS version 20 (Chicago, IL, USA) for analysis. Both descriptive and inferential statistics were performed. Frequencies, proportion and summary statistics were used to describe the study population in relation to relevant variables. The degree of associations between malaria cases and selected risk factors were computed using logistic regression analysis together with their corresponding 95% confidence interval.

The study obtained ethical approval from the Institutional Review Board of Dilla University. Permission to conduct the study was also obtained from the health bureau director office. Only children who assented and whose parents or guardians consented to involve in the study.

## Results

Overall, 324 individuals were included with 100% response rate. One hundred two (31.4%) of the participants were in the age group of 15-24 yr while 173 (53.4%) of individuals were male. The majority of the respondents (25%) were in the grade level of 9-12. One hundred seventy one (52.8%) of the participants had a family size of 4 and below persons per head and most of them (68.2%) had monthly income of <45 US$ (Table 1).

**Table 1:** Socio-demographic characteristics of the study subjects (n=324)

**Variables**	**Frequency**	**Percent**
Age groups (yr)		
0-4	11	3.4
5-14	83	25.6
15-24	102	31.4
25-34	79	24.4
35-44	17	5.3
45-54	19	5.9
>55	13	4.0
Sex		
Male	173	53.4
Female	151	46.6
Family size		
<4	171	52.8
≥4	153	47.2
Years of educational		
Illiterate	49	15.1
1-4	59	18.2
5-8	62	19.1
9-12	81	25.0
>12	73	22.5
Monthly income (UD$)		
<45	221	68.2
≥45	103	31.8

**Figure 1:**
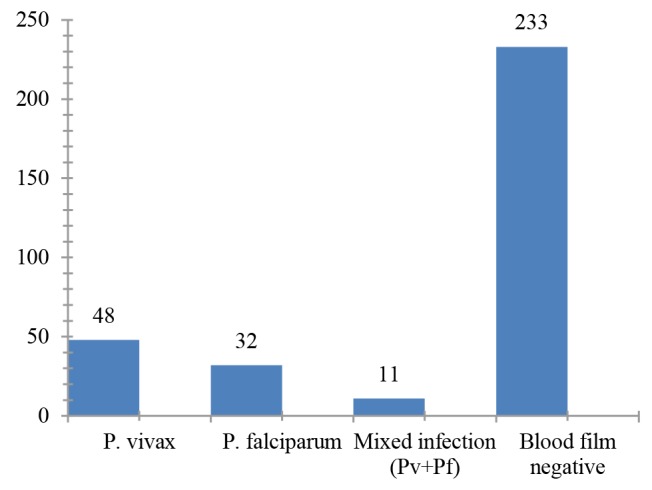
Frequency of malaria infection by parasite species among the study subjects

The overall prevalence of malaria infection in the study area was 91(28.1%). The species wise distribution showed *P. vivax* was the predominant species, accounting for 48 (52.7%), followed by *P. falciparum* 32(35.2%) and mixed infection with both *P. vivax* and *P. falciparum* 11(12.1%) (Figure 1).

Regarding malaria cases with respect to the housing condition of the subjects, (Table 2), getting chance of malaria infection was significantly higher when the wall of the house was mud blocks/bricks, house floor was local dung and when living in stick and mud roofs.

**Table 2:** Logistic regression analysis for the association malaria parasites with the housing construction of the study participants

**Characteristics**	**Malaria cases** **n=91**	**Malaria negative** **n=233**	**Odds ratio** ** (95% CI)**
Main material in walls	
Sticks and wood planks	17	40	1.00
Mud blocks/bricks	50	92	6.57 (5.33, 49.22)
Cement blocks/bricks	24	101	3.13 (1.21, 11.16)
Main material in roof		
Corrugated iron	17	44	1.00
Thatch	20	85	6.33 (4.49, 31.91)
Stick & mud	54	104	8.39 (5.04, 30.27)
Main material in floor		
Cement	7	31	1.00
Earth/sand	39	96	1.27 (1.07, 2.06)
Local dung	45	106	1.70 (1.20, 3.45)

Logistic regression analysis findings showed that participants whose house had been sprayed with insecticide in the past 6 months were three times less likely to get malaria infection (OR = 0.33, 95% CI: 0.11, 0.92). Approximately two fold increased prevalence of malaria was observed in individuals’ who had been living in the nearby stagnant water than those who were living far away (by at least 1 km away from their residence) from these risky areas (OR=1.87, 95% CI: 1.20, 2.76). Individuals having and using insecticide treated nets for the last six months were 0.2 times less likely to get malaria parasites than those of not having adequate bed nets (OR=0.19, 95% CI: 0.09, 0.37). Similarly, participants accessing health facilities were 0.3 times less likely to be infected by malaria parasites than those who had no access (OR=0.32, 95% CI: 0.29, 0.85) (Table 3).

**Table 3:** Logistic regression analysis of the association of different variables with malaria parasites

**Variables**	**Malaria cases** **n=91**	**Malaria negative** **n=233**	**Odds ratio** **(95% CI)**
Sex			
Female	40	111	1.00
Male	51	122	1.12 (0.63, 2.00)
Presence of stagnant water		
No	23	94	1.00
Yes	68	139	1.87, (1.20, 2.76)
Bed net utilization		
No	50	195	1.00
Yes	41	38	0.19, (0.09, 0.37)
Insecticide spray		
No	16	21	1.00
Yes	75	212	0.33, (0.11, 0.92)
Family size			
>5	34	60	1.00
<5	57	173	1.13, (0.72, 2.03)
Monthly income (UD$)		
<45	62	159	1.00
≥45	29	74	1.38(0.54, 3.55)
Availability of health services	
No	23	25	1.00
Yes	68	208	0.32, (0.29, 0.85)

## Discussion

Malaria infection is high in the area among the selected population. A large proportion of the infected individuals were male, and 52.75% of the cases were *P. vivax* infection. The logistic regression analysis showed that the infection was associated with different factors.

The finding of the current study revealed that malaria infection in the study participants was 91(28.1%). This report is comparable with the study conducted among patients attending public health facilities of Maputo City, Mozambique with 15.7% of prevalence^20^. Similarly, the result from Hadhramout, Yemen visited to health facilities 18.8%^21^ and from Hadiya, Ethiopia^16^ with 25.8%. The current report was higher than reports from Arsi Negele Ethiopia^22^, Butajira Ethiopia^23^, and Bangladesh^24^ with 11.45%, 0.93%, 3.97% and 2.2% respectively. In contrary, it is less than that reported in Kersa Woreda Jimma 43.8%^25^, Hallaba, Ethiopia^26^ with 82.8% and Nigeria by 39.5%^27^. The observed difference might be due to the seasonality of malaria and the different control measures in the current study area for the previous years.

The species wise distribution shows *P. vivax* was the predominant species, accounting for 48(52.7%), followed by *P. falciparum* 32(35.2%) and mixed infection with both *P. vivax* and *P. falciparum* 11(12.1%). This is in line with the studies conducted in Arsi Negelle Health Center, Ethiopia^22^ and Hallaba, Southern Ethiopia^26^. However, this finding is disagreeing with the national prevalence indicating of *P. falciparum* and *P. vivax*, which is 65%-75% and 25%-35% respectively^3^. In addition, studies conducted in hospitals, Islamabad, Pakistan^28^ and Hadhramout, Yemen^21^ are not supporting the present finding, reporting a higher prevalence of *P. falciparum* infection. The deviation between the finding of this study and the national figure of epidemic regarding *P. vivax* along with *P. falciparum* might be due to relapsing by *P. vivax*. A more than two-fold increase in the rate of drug resistance has been reported in south-central Ethiopia, close to the study area^29^. Thus, chloroquine resistant *vivax* malaria may have accounted for the dominance of *vivax* observed in the current study.

Concerning gender-wise prevalence of malaria, the cases were high among males with 51(56.04%), but did not show significant association. This is consistent with the findings of Ayele ET AL.^14^, Ferede et al.^30^ and Alemu et al. ^31^ who reported infection rate is higher among males in Northwest Ethiopia but differs from the findings of Tefera^26^ in similar study, in Ethiopia, where female subjects were more infected.

It is observed that rate of infection with the disease was significantly higher when the wall of the house was mud blocks/bricks; house floor was local dung and when living in stick and mud roofs. This is similar with some of the findings from previous reports^14^. This is because it is most suited for porous surfaces such as brick, thatch, dung and mud walls for residence of mosquito vectors.

Moreover, participants whose house has been sprayed with insecticide with the currently supplied insecticides by the government (Proxus and Bifenthrin) in the past 6 months are three times less likely to get malaria infection (OR = 0.33, 95% CI: 0.11, 0.92). This is similar with the studies done in East Shoa Zone, Ethiopia^32^ and Maputo City, Mozambique^20^. Approximately twofold increased prevalence of malaria was observed in individuals’ who have been living in the nearby stagnant water than those who were far away (by at least 1 km away from their residence places) from these risky areas (OR=1.87, 95% CI: 1.20, 2.76). Living in the nearby stagnant water was also identified as a risk factor. The significantly higher parasite rate was found among the individuals having stagnant water in their compound. It can be explained from the fact that they are more exposed to mosquito bites, because these areas are suitable for breeding of mosquitoes around their homes. Individuals having and using insecticide treated nets for the last six months were around 0.2 times less likely to get malaria parasites than those of not having adequate bed nets (OR=0.19, 95% CI: 0.09, 0.37). Similarly, participants accessing health facilities were 0.3 times less likely to be infected by malaria parasites than those who have no access (OR=0.32, 95% CI: 0.29, 0.85). This finding is in agreement with the reports of Nahum et al.^33^ with (OR=0.77; 95% CI =0.61, 0.97).

There were some limitations in our study. The study was conducted at one point in time and did not redirect the yearly seasonal pattern of the disease. Since, it is possible that seasonal variation will have affected the study findings. Malaria cases in the area could vary with the seasons, rainfall patterns and years. Next, the finding is not representative of the whole community since data was collected from only those individuals who came to health centres for health services.

## Conclusions

Most of the infected participants were male with high prevalence of *P. vivax* species. There was a significant association of malaria cases with the type of housing construction, bed net ownership, presence of mosquito breeding places and insecticide spray of the respondents’ houses. Further studies needed to be conducted for the status and contributing factors of epidemiological shifting of malaria parasites in the study area in large scale.

## Acknowledgments

We would like to forward our appreciation to Dilla University, Research and Dissemination Office for financial and material support of the research.

## Conflict of interest statement

The authors declared that no competing interests exist regarding the publication of this paper.

## Highlights

- Prevalence of malaria is high in the study area .
- Plasmodium vivax cases are more prevalent than Plasmodium falciparum.
- Type of housing construction and bed net use showed association with malaria infection.
- Malaria infection did not show significant association with gender and monthly income.
